# FADD as a key molecular player in cancer progression

**DOI:** 10.1186/s10020-022-00560-y

**Published:** 2022-11-08

**Authors:** Ying Liu, Xiaoge Li, Xuehao Zhou, Jianxun Wang, Xiang Ao

**Affiliations:** 1 Institute for Translational Medicine, The Affiliated Hospital of Qingdao University, Qingdao Medical College, Qingdao University, 266021 Qingdao, China; 2 School of Basic Medical Sciences, Qingdao Medical College, Qingdao University, 266071 Qingdao, China

**Keywords:** FADD, Inflammation, Drug resistance, Biomarker, Therapeutic target

## Abstract

Cancer is a leading disease-related cause of death worldwide. Despite advances in therapeutic interventions, cancer remains a major global public health problem. Cancer pathogenesis is extremely intricate and largely unknown. Fas-associated protein with death domain (FADD) was initially identified as an adaptor protein for death receptor-mediated extrinsic apoptosis. Recent evidence suggests that FADD plays a vital role in non-apoptotic cellular processes, such as proliferation, autophagy, and necroptosis. FADD expression and activity of are modulated by a complicated network of processes, such as DNA methylation, non-coding RNA, and post-translational modification. FADD dysregulation has been shown to be closely associated with the pathogenesis of numerous types of cancer. However, the detailed mechanisms of FADD dysregulation involved in cancer progression are still not fully understood. This review mainly summarizes recent findings on the structure, functions, and regulatory mechanisms of FADD and focuses on its role in cancer progression. The clinical implications of FADD as a biomarker and therapeutic target for cancer patients are also discussed. The information reviewed herein may expand researchers’ understanding of FADD and contribute to the development of FADD-based therapeutic strategies for cancer patients.

## Background

Cancer, one of the most prevalent malignant diseases, is the second leading cause of death around the world (Liu et al. 2022f). According to the latest data from the GLOBOCAN project and the United Nations, there will be approximately 4,820,000 and 2,370,000 new cancer diagnoses and almost 3,210,000 and 640,000 cancer-related deaths in China and the United States in 2022, respectively (Xia et al. 2022). In recent years, the incidence and mortality of cancer have gradually increased due to rapid population aging and the accumulated effects of risk factor exposure. Despite significant advances in therapeutic interventions, cancer remains a major threat to human health due to its highly lethal features and high recurrence rate (Wang et al. 2022; Zuo et al. 2022). Clinically, the majority of cancer patients are routinely diagnosed at a relatively advanced stage, with a poor five-year survival (Wang et al. 2021; Zhang et al. 2022). This is mainly caused by limited knowledge regarding the pathogenesis of cancer, lack of effective diagnosis and treatment methods, and the development of therapy resistance. Therefore, there is great urgency in clarifying the mechanisms involved in cancer progression as well as identifying effective therapeutic targets and screening diagnostic and prognostic biomarkers with high specificity and sensitivity in cancer treatment.

Fas-associated protein with death domain (FADD), also known as MORT-1, is an evolutionarily conserved death domain (DD)-containing cytoplasmic adaptor protein and is widely expressed in various species (Mouasni and Tourneur 2018). FADD was initially reported as an adaptor molecule that bridges activated death receptors (DRs) with initiator caspase to mediate DR-induced extrinsic apoptosis (Lee et al. 2012b; Sprick et al. 2000). It is also a key modulator in non-apoptotic cellular processes, such as proliferation, cell cycle, and autophagy (Alappat et al. 2003; Antunovic et al. 2019; Imtiyaz et al. 2009). FADD has been shown to play a vital role in a variety of physiological and pathological processes, including embryonic development, innate immunity, and inflammation (Lee et al. 2012b). Therefore, the dysregulation of FADD is closely correlated with many diseases, particularly cancer (Debnath et al. 2022; Lin et al. 2020; Meer et al. 2022). The aberrant expression of FADD has been observed in multiple solid tumors, such as glioma, non-small cell lung cancer (NSCLC), and hepatocellular carcinoma (HCC) (Chen et al. 2021; Han et al. 2020; Harari-Steinfeld et al. 2021). FADD dysregulation contributes to cancer progression by affecting many aspects of cancer cell behavior, including proliferation, apoptosis, cell cycle, autophagy, inflammation, and drug resistance (Marin-Rubio et al. 2019b; Mrkvova et al. 2021). However, the exact FADD mechanisms involved in cancer progression are still not fully understood. In addition, due to its crucial roles and differential expression patterns, FADD has exhibited great potential as a valuable therapeutic target and biomarker for cancer diagnosis and treatment prognosis.

In this review, we mainly summarize recent findings on the structure, functions, and regulatory mechanisms of FADD, focus on its mechanisms of action in cancer progression, and highlight its clinical implications in cancer treatment. We also discuss future research directions aimed at designing FADD-based therapeutic strategies for cancer patients.

## Structure and functions of FADD

### Structural features of FADD

**Fig. 1:**
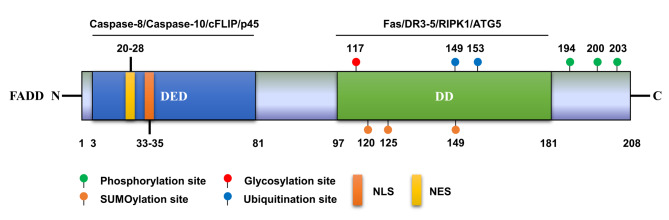
Protein structure and PTM sites of human FADD. The structural domains are presented in the bar. The well-known PTM sites are indicated at the corresponding position. The proteins interacting with FADD are exhibited above the lines at the corresponding domains. PTM, post-translational modification; FADD, Fas-associated protein with death domain; DED, ; DD, death effector domain; NLS, nuclear localization signal; NES, nuclear export signal; cFLIP, cellular FLICE (caspase 8)-inhibitory protein; DR, death receptor; RIPK1, receptor-interacting serine/threonine protein kinase 1; ATG5, autophagy related-protein 5

The *FADD* gene is mapped to the human chromosomal band 11q13 and encodes a functional FADD protein (about 23 kDa) composed of 208 amino acids. The structure of FADD is evolutionarily conserved across species and contains two main domains, the C-terminal DD and the N-terminal death effector domain (DED) (Fig. 1). The DD (amino acids 97 to 181) of FADD mainly mediates its binding to the DRs (e.g., Fas and DR3-5) of the tumor necrosis factor (TNF) receptors superfamily to transmit apoptosis-initiating signals (Marin-Rubio et al. 2019b). Moreover, the DD mediates the interaction of FADD with receptor-interacting protein kinase (RIPK) 1 and autophagy related-protein 5 (ATG5), thereby inducing necrosis and autophagy (Pyo et al. 2005; Vanden Berghe et al. 2004). The DED also contains a nuclear localization signal (NLS) and a nuclear export signal (NES) that determine the translocation of FADD between the nucleus and cytoplasm (Gomez-Angelats and Cidlowski 2003). The DED (amino acids 3 to 81) is primarily responsible for the interaction of FADD with caspases (e.g., caspase-8 and 10) to form the death-inducing signal complex (DISC) (Bhojani et al. 2005). The DED of FADD could also mediate its binding to the cellular FLICE (caspase 8)-inhibitory protein (cFLIP) in promoting cellular proliferation (Vesely et al. 2009). Furthermore, the DED was found to mediate the interaction of FADD with p45 to diminish Fas-FADD-mediated death signaling (Sung et al. 2013).

### Functions of FADD

FADD is a well-studied cytoplasmic adaptor protein that transmits DR-mediated extrinsic apoptotic signals (Zhou et al. 2022b). It can act as a bridge to interact with DRs and caspases-8/10 via its DD and DED, respectively, thereby forming an active DISC. In the DISC, caspases-8/10 are activated and then initiate the caspase cascade by a procaspase-3 or BID cleavage, eventually inducing apoptosis (Zhang et al. 2008). FADD is also involved in other forms of cell death. It has been identified as a negative regulator of necroptosis, a passive and traumatic death mediated by a RIPK1/RIPK3/mixed lineage kinase domain-like protein (MLKL) axis. FADD reduces the activity of MLKL by inducing a cleavage of the RIPK1/RIPK3 kinase domain by recruiting caspase-8/cFLIP, finally resulting in the inhibition of necroptosis (Lin et al. 1999; Osborn et al. 2010). Furthermore, FADD also plays a role in autophagic cell death and has been found to mediate the promotion of ATG5 on the autophagic death of HeLa cells triggered by interferon (IFN)-γ. FADD knockdown in HeLa cells suppresses ATG5-mediated cell death, leading to a switch from apoptotic signaling to necroptosis (Pyo et al. 2005). Some evidence suggests that FADD is involved in the regulation of several cellular processes, including cell proliferation, survival, and cell cycle (Hollomon et al. 2020; Wang et al. 2017). In addition, it has emerged as a component of various signalosomes, such as necrosome, innateosome, and inflammasome, strongly indicating the essential nature of its role in innate immunity and inflammation.

Structural biology is a hot research field in science that deeply uncovers the biomolecule structures and their structure-function relationships (Mihasan et al. 2019). With the rapid development of FADD structural biology, its functional characteristics are being elucidated from a higher-level vision in recent years. Cryogenic electron microscopy (cryo-EM) is a mainstream tool in structural biology, aimed at efficiently determining high-resolution structure of protein (Liu et al. 2022 g). Fox et al. analyzed the structure of FADD/Caspase-8 complexes using cryo-EM. They found that FADD-nucleated tandem DED (tDED) helical filament orientated the procaspase-8 catalytic domains, thereby activating them through anti-parallel dimerization. The resultant catalytically active FADD/Caspase-8 complex triggered cellular apoptosis. Moreover, c-FLIPS could be recruited into this complex and decrease Caspase-8 activity by altering tDED triple helix architecture (Fox et al. 2021). Eberstadt et al. analyzed the three-dimensional solution structure of a soluble, biologically active mutant of FADD DED using Nuclear Magnetic Resonance, a powerful method for detecting protein structure, interaction, and dynamics at atomic resolution. The structure consisted of six antiparallel, amphipathic α-helices, and the hydrophobic region of FADD DED involving F25 was vital for its binding to FLICE and inducing apoptosis (Eberstadt et al. 1998).

Collectively, these studies strongly suggest that FADD is a key regulators in a variety of physiological and pathological processes. Currently, an increasing number of studies suggest that FADD dysregulation contributes to cancer progression. However, further research is required to elucidate the underlying mechanisms of FADD involved in cancer progression, which may be of great benefit to the development of FADD-based therapeutic strategies for cancer patients.

### Molecular mechanisms of FADD regulation

**Fig. 2:**
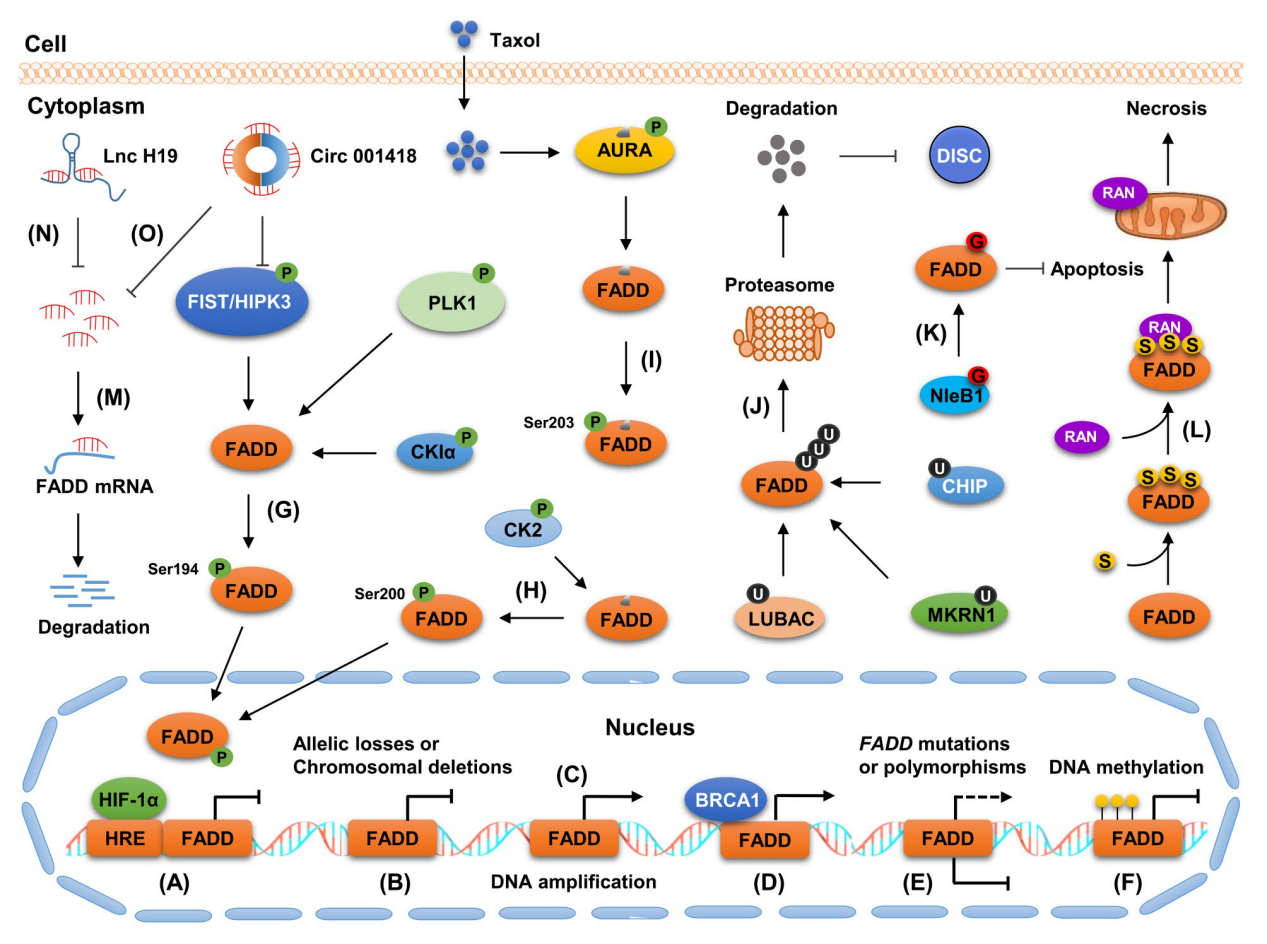
Regulation of FADD. FADD expression and activity are regulated by distinct mechanisms, including genetic and chromosomal alterations, DNA methylation, TFs, ncRNAs, and PTMs. (A-F) HIF-1α, allelic losses or chromosomal deletions, DNA amplification, BRCA1, gene mutations or polymorphisms and DNA methylation alter FADD expression and activity at the transcription level. (G) FIST/HIPK3, PLK1, and CKIα facilitate the translocation of FADD from the cytoplasm to the nucleus by mediating its phosphorylation at Ser194. (H) CK2 promote the nuclear localization by mediating its phosphorylation at Ser200. (I) AURA phosphorylates FADD at Ser203 in response to Taxol exposure. (J) LUBAC, MKRN1, and CHIP inhibit the formation of DISC by facilitating the degradation of FADD in an ubiquitin-proteasome dependent manner. (K) NleB1 promoted the GlcNAcylation of FADD at Arg117, thereby antagonizing the DR-induced apoptosis. (L) SUMOylation of FADD facilitates the recruitment of Drp1 onto the mitochondria by enhancing its binding to Drp1, resulting in mitochondrial-fragmentation-associated necrosis. (M) MiRNA promotes the degradation of FADD mRNA by directly targeting its 3′UTR. (N) H19 downregulates FADD by sponging miR-675. (O) Circ 001418 increased FADD levels by targeting miR-1297

FADD expression and activity are modulated by distinct mechanisms, including genetic and chromosomal alterations, DNA methylation, transcription factors (TFs), non-coding RNAs (ncRNAs), and post-translational modifications (PTMs) (Fig. 2). Herein, we present a summary of the main modes of FADD regulation, focusing specifically on the mechanisms of FADD regulation in cancer.

### Genetic and chromosomal alterations contribute to FADD regulation

DNA amplification is one of the major events resulting in high FADD expression. FADD-containing DNA amplification of the 11q13 region has been observed in many types of cancer, including breast cancer (BC), bladder cancer (BCa), laryngeal/pharyngeal cancer, oral squamous cell carcinoma (OSCC), lung squamous cell carcinoma (LUSC), ovarian cancer (OC), esophageal squamous cell carcinoma (ESCC), and head and neck squamous cell carcinoma (HNSCC) (Brown et al. 2008; Chien et al. 2016; Eytan et al. 2016; Gibcus et al. 2007; Katoh and Katoh 2005; Petersen et al. 2000; Qin et al. 2016; Zhou et al. 2022a). Among them, the amplification of the *FADD* gene is most prominent in ESCC and HNSCC, with approximately 60% and 30% of patient samples harboring the amplification, respectively (Marin-Rubio et al. 2019b). Allelic losses or chromosomal deletions may result in low FADD expression. Loss of heterozygosity and deletions encompassing the *FADD* gene have been observed in 25% of cervical cancer (CC) samples (Srivatsan et al. 2002). In addition, mutations and polymorphisms in *FADD* gene have also been reported (Soung et al. 2004; Villa-Morales et al. 2010), indicating that they may also alter FADD expression and activity.

### FADD regulation at the transcription level

DNA methylation is a classical mechanism that regulates gene expression. Promoter hypermethylation suppresses gene expression at the transcriptional level, whereas hypomethylation plays the opposite role (Liu et al. 2019). *FADD* promoter hypermethylation has been found in some diseases, such as apical periodontitis, myelodysplastic syndrome, and OSCC. Correspondingly, low FADD expression has been observed in these diseases (Saberi et al. 2014; Wichnieski et al. 2019; Zhao et al. 2019). Moreover, FADD is a downstream target gene of several TFs, such as the hypoxia-inducible factor-1α (HIF-1α) and the BC susceptibility gene 1 (BRCA1). Hindryckx et al. discovered that *FADD* promoter contained a hypoxia-responsive element (HRE). Functional analysis revealed that HIF-1α inhibited the transcriptional activity of *FADD* gene in colon cancer cells by directly binding to HREs in its promoter. Consistent with this, the deletion of the core nucleotides in the HRE of *FADD* promoter resulted in the loss of HIF-1α-mediated transcriptional activity suppression (Hindryckx et al. 2010). In another study, Nguyen et al. showed that BRCA1 overexpression in BRCA1-deficient BC cells significantly upregulated FADD expression by directly binding to *FADD* promoter. By contrast, BRCA1 knockdown efficiently decreased FADD expression at both the protein and messenger RNA (mRNA) levels (Nguyen et al. 2018).

### Post-transcriptional regulation of FADD by ncRNAs

NcRNAs are a group of regulatory RNA molecules of different sizes, structures, and biological functions, with limited or no protein-coding capacity (Wang et al. 2020; Wen et al. 2021; Zhang et al. 2021c). They play a vital role in almost all biological processes by modulating the expression of specific gene targets (Zhang et al. 2020a, c, 2021b). NcRNAs can be divided into distinct categories, including microRNAs (miRNAs), long non-coding RNAs (lncRNAs), circular RNAs (circRNAs), and piwi-interacting RNAs (piRNAs), based broadly on their size and function(Chang et al. 2022a, b; Jia et al. 2022; Liu et al. 2021a, b; Zhang et al. 2021a). Recent studies have demonstrated that FADD expression is regulated by a series of ncRNAs in a variety of diseases (Table 1).

**Table 1:** NcRNAs targeting FADD in disease

Disease types	ncRNAs	Key messages	References
Cancer	miR-128a	MiR-128a overexpression enhanced Fas-resistance on Jurkat cells by directly targeting FADD. MiR-128a knockdown sensitized Jurkat/R cells to the Fas-induced apoptosis through releasing FADD expression.	Yamada et al. (Yamada et al. 2014)
	miR-633	MiR-633 inhibited FADD expression by directly targeting its 3’UTR, leading to the enhancement of DOX/CDDP resistance in GC.	Pang et al. (Pang et al. 2019)
	H19 miR-675	Overexpression of miR-675 derived from lncRNA *H19* promoted liver necroptosis and cell death in HCC cells by targeting FADD.	Harari-Steinfeld et al. (Harari-Steinfeld et al. 2021)
		Overexpression of H19 and miR-675 facilitated proliferation and suppressed apoptosis in GC cells by inhibiting the caspase cleavage cascades (caspase 8 and caspase 3) via downregulating FADD.	Yan et al. (Yan et al. 2017)
		Overexpression of H19 increased PTK2 levels by targeting miR-138 and decreased FADD levels by targeting miR-675, leading to the inhibition of cell apoptosis and promotion of hepatoblastoma carcinogenesis.	Ge et al. (Ge et al. 2019)
	circ 001418 miR-1297	Overexpression of circ 001418 facilitated cell proliferation and transfer, and reduced apoptosis by upregulating EphA2 and cytochrome c protein expression, and downregulating FADD via sponging miR-1297.	Peng et al. (Peng et al. 2021)
Myocardial infarction	miR-103	Inhibition of miR-103 significantly improved the myocardial infarction of mice by releasing FADD.	Zaafan et al. (Zaafan and Abdelhamid 2021)
	H19 miR-103/107	FADD was upregulated by H19 via sponging miR-103/107. FADD upregulation promoted necrosis and improved myocardial infarction by influencing the formation of RIPK1 and RIPK3 complexes.	Wang et al. (Wang et al. 2015)
Atherosclerosis	miR-155	MiR-155 reduced the macrophage apoptosis by downregulating FADD via directly binding to its 3’-UTR region.	Zhu et al. (Zhu et al. 2013)
Intervertebral disc degeneration	miR-155	MiR-155 facilitated Fas-mediated apoptosis of nucleus pulposus cells by targeting FADD and caspase-3 via directly binding to their 3’-UTRs.	Wang et al. (Wang et al. 2011a)

MiRNAs are short single-stranded ncRNAs with 18–25 nucleotides in length (Zhang et al. 2018). Their canonical role is to suppress the expression of specific target genes by directly binding to the 3′-untranslated region (3′UTR) of the mRNA (Zhang et al. 2020b; Zhou et al. 2022c). FADD is a target of miRNAs. In our previous work, we showed that high miR-633 expression was closely associated with FADD loss in gastric cancer (GC) tissue and cell lines. The knockdown of miR-633 significantly upregulated FADD in GC cells. Functional analysis revealed that miR-633 significantly decreased FADD levels by directly targeting the 3′UTR of its mRNA, resulting in the enhancement of doxorubicin/cisplatin (DOX/CDDP) resistance in GC (Pang et al. 2019). In another study, we found that miR-103/107 contributed to necrotic cell death of H9c2 cardiomyocytes by directly targeting FADD. Consistent with this, miR-103/107 knockdown blocked necrosis in a cellular model as well as myocardial infarction in a mouse ischemia/reperfusion model (Wang et al. 2015). Furthermore, FADD was identified as a direct target of miR-155 in nucleus pulposus (NP) cells. MiR-155 inhibited FADD expression by directly targeting its 3’UTR, thereby promoting Fas-mediated apoptosis in human intervertebral disc degeneration (Wang et al. 2011a). In addition, FADD expression was modulated by a series of miRNAs, such as miR-675, miR-7a, and miR-128a, in several types of cancer (Ge et al. 2019; Liu et al. 2016; Yamada et al. 2014). LncRNAs and circRNAs are crucial regulators of FADD. For instance, lncRNA H19 was found to decrease FADD levels by targeting miR-675 in GC, HCC, and liver necroptosis (Ge et al. 2019; Harari-Steinfeld et al. 2021; Yan et al. 2017). Circ 001418 upregulated FADD by targeting miR-1297 in BCa (Peng et al. 2021).

Taken together, these studies strongly indicate that FADD is tightly controlled by an ncRNA-based network. The dysregulation of FADD induced by this network may contribute to the development many diseases, particularly cancer. Therefore, a complete clarification of the ncRNA-based regulatory network of FADD may be highly beneficial to the development of effective therapeutic strategies for cancer patients.

### Importance of PTMs in modulating FADD

PTMs are dynamic and reversible processes occurring in protein during or after their synthesis. They play a vital role in modulating protein functions such as subcellular localization, stability, and activity Liu et al. 2022c; Liu et al. 2022 h). Studies have increasingly demonstrated that FADD can be modified by various PTMs, such as phosphorylation, ubiquitination, SUMOylation, and GlcNAcylation. FADD was found to be a substrate of multiple kinases, including FIST/HIPK3 kinase, casein kinase (CK)Iα, polo kinase (PLK)1, Aurora-A kinase (AURA), and CK2 (Alappat et al. 2005; Jang et al. 2011a, b; Rochat-Steiner et al. 2000; Sheikh and Huang 2003; Vilmont et al. 2015). Distinct kinases can phosphorylate FADD at diverse sites and generate a range of cellular effects, and FADD can be phosphorylated by FIST/HIPK3, CKIα, and PLK1 at Ser194 (Alappat et al. 2005; Jang et al. 2011b; Rochat-Steiner et al. 2000). The phosphorylation of this site mediated the translocation of FADD between the cytoplasm and the nucleus (Sheikh and Huang 2003). AURA directly phosphorylated FADD at Ser203 in response to Taxol treatment (Jang et al. 2011a). Moreover, FADD was phosphorylated by CK2 at Ser200, which was required for its nuclear localization (Vilmont et al. 2015). Ubiquitination is a crucial PMT that mediates the degradation of proteins in a proteasome-dependent manner. Currently, several E3 ubiquitin ligases for FADD have been identified, including the makorin ring finger protein 1 (MKRN1), the C terminus HSC70-interacting protein (CHIP), and the linear ubiquitin chain assembly complex (LUBAC) (Goto and Tokunaga 2017; Lee et al. 2012a; Seo et al. 2018). Lee et al. discovered that MKRN1 promoted the ubiquitin-dependent degradation of FADD. MKRN1 knockdown enhanced FADD stability and facilitated DISC formation, thereby causing hypersensitivity to extrinsic apoptosis (Lee et al. 2012a). Seo et al. revealed that CHIP inhibited DISC formation and apoptosis in HeLa cells by inducing the polyubiquitylation of FADD at Lys149 and 153. Consistent with this, CHIP deficient in E3 ligase activity failed to suppress the function and ubiquitylation of FADD (Seo et al. 2018). Moreover, FADD was a substrate of LUBAC, which mediated its linear ubiquitination upon TNFα stimulation in T-cell leukemia Jurkat cells (Goto and Tokunaga 2017).

SUMOylation is a type of ubiquitination-like PTM that plays a vital role in the modulation of protein function (Fan et al. 2022). FADD was found to be modified by SUMO2 at Lys120/125/149 during intrinsic necrosis of HeLa cells. SUMOylation promoted dynamin-related protein 1 (Drp1) recruitment onto the mitochondria by enhancing the binding of FADD to Drp1, resulting in Drp1-mediated mitochondrial fragmentation. Consistent with this, SUMO-defective FADD expression in murine embryonic fibroblasts (MEFs) blocked mitochondrial-fragmentation-associated necrosis (Choi et al. 2017). In addition, FADD is a substrate of NleB1, an arginine glycosyltransferase that modifies conserved arginine residues in DD-containing host proteins with N-acetylglucosamine (GlcNAc). NleB1 promoted the GlcNAcylation of FADD at Arg117 in HeLa cells during bacterial infection, thereby antagonizing the DR-induced apoptosis of infected cells (Scott et al. 2017). Collectively, these findings suggest that FADD activity is regulated by various PTMs. Thus in-depth investigations are required to further explore the PTM network involved in FADD regulation.

### Implications of FADD in cancer progression

FADD was deficient in certain types of cancer, such as thymic lymphoma, acute myeloid leukemia (AML), and glioblastoma (GBM) (Newton et al. 2000; Tourneur et al. 2004; Wang et al. 2017). However, high FADD expression was also observed in multiple cancer types, including pancreatic cancer (PC), laryngeal/pharyngeal cancer, OSCC, HNSCC, and BC (Chien et al. 2016; Gibcus et al. 2007; Gonzalez-Moles et al. 2020; Zhang et al. 2017; Zhou et al. 2022a). These findings strongly suggest that FADD may play a dual role in cancer progression by acting as a tumor suppressor or oncoprotein. However, the exact mechanisms are yet to be fully understood. In the following section, we summarize the recent advances regarding the role of FADD in cancer progression (Table 2).

**Table 2:** Functional roles of FADD in different types of cancer

Cancer types	Key messages	References
BC	High expression or amplification of *FADD* was negatively associated with the abundance of CD4 + T cells and dendritic cell infiltration in BC (p < 0.05). High expression of *FADD* mRNA significantly associated with RFS BC in patients (p < 0.05). High expression of FADD was frequently detected in luminal B and high-grade BC with shorter metastasis-free survival times (p < 0.05).	Zhou et al. (Zhou et al. 2022a)
	Increase copy number and mRNA overexpression of FADD are frequently observed in primary BC.	Callegari et al. (Callegari et al. 2016)
	FADD knockdown promoted autophagy of BC cells by downregulating Rheb and inhibiting mTORC1 activity.	He et al. (He et al. 2016)
	FADD expression was significantly associated with T stage (P = 0.046). The combination of *FADD*, *PPFIA1*, and *TMEM16A* genes was significantly correlated with perineural invasion (p = 0.022) and DFS (p = 0.034).	Choi et al. (Choi et al. 2014)
	MKRN1 mediated the ubiquitination and proteasomal degradation of FADD. The stabilization of FADD facilitated extrinsic apoptosis by promoting caspase-8 and caspase-3 cleavage in BC cells. FADD participated in the modulation of necroptosis upon caspase inhibition.	Lee et al. (Lee et al. 2012a)
	JNK-mediated phosphorylation of FADD inhibited cell growth and metastasis through G2/M arrest.	Matsuyoshi et al. (Matsuyoshi et al. 2006)
GC	FADD was negatively regulated by miR-633, and its upregulation enhanced DOX/DDP induced apoptosis in GC cells.	Pang et al. (Pang et al. 2019)
	FADD expression was negatively associated with H19 and miR-675 expression in GC. FADD mediated the promotion of H19/miR-675 axis on GC progression.	Yan et al. (Yan et al. 2017)
	The expression of p-FADD in GC was heterogenous in its location, whereas its expression in normal gastric cells was uniform nuclear expression.	Yoo et al. (Yoo et al. 2007)
NSCLC	NSCLC patients with overexpression of FADD had lower OS rates (p = 0.033). The upregulation of FADD was an independent poor prognostic biomarker for patients with surgically resected NSCLC (p = 0.027).	Chen et al. (Chen et al. 2021)
	High FADD expression predicted poor prognosis of NSCLC patients. The ubiquitin ligase SPOP promoted FADD degradation by directly binding to it. FADD mediated the inhibition of SPOP on NF-κB activity and its target genes expression.	Luo et al. (Luo et al. 2018)
	FADD was specifically downregulated in NSCLC cells, and FADD loss was significantly associated with the presence of extracellular FADD. NSCLC tissues released significantly more FADD than non-tumoral tissue (P = 0.000003). The release of FADD increased significantly with the cancer stage, and was associated with both early and late steps of the metastasis process.	Cimino et al. (Cimino et al. 2012)
PCa	PCa patients with a greater positive p-FADD rate had a significantly lower biochemical recurrence rate than those with a lower positive p-FADD rate (p < 0.001). The positive p-FADD rate was negatively associated with Gleason score.	Ikeda et al. (Ikeda et al. 2013)
	The overexpression of dephosphorylated FADD (S194A) promoted proliferation and invasion of PCa cells by enhancing hTERT expression and telomerase activity. PCa patients expressing low levels of p-FADD had significantly higher rates of biochemical recurrence than those with high p-FADD expression (p < 0.001).	Matsumura et al. (Matsumura et al. 2009)
	The overexpression of phosphorylated FADD resulted in G2/M cell-cycle arrest, whereas the non-phosphorylated FADD overexpression resulted in cell cycle progression and enhanced colony-forming activity in PCa cells.	Shimada et al. (Shimada et al. 2005)
	FADD was phosphorylated by JNK at Ser194 in PCa cells treated with PTX. Phosphorylated FADD enhanced the synergistic effects of paclitaxel on anticancer drug-induced apoptosis in PCa cells.	Shimada et al. (Shimada et al. 2004)
	FADD mediated the effect of PTEN in promoting drug-induced apoptosis by facilitating caspase-8 activation and BID cleavage in PCa.	Yuan et al. (Yuan and Whang 2002)
Leukemia	FADD knockdown in Jurkat cells significantly inhibited cell proliferation, enhanced sensitivity to Etoposide-induced intrinsic apoptosis and resistance to TRAIL-induced extrinsic apoptosis by triggering a metabolic shift from glycolysis to mitochondrial respiration.	Zhou et al. (Zhou et al. 2022b)
	FADD knockdown in Jurkat cells enhanced drug resistance, which could be partially overcome by induction of RIP1-dependent necroptosis through TNFR1 activation using combined treatment with TNFα and LCL161.	Mrkvova et al. (Mrkvova et al. 2021)
	S194-P-FADD was more stable and preferentially localized to the cell nucleus, thereby promoting proliferation in T-cell lymphoblastic lymphoma cells. FADD phosphorylation was a prognostic biomarker in T-cell lymphoblastic lymphoma.	Marin-Rubio et al. (Marin-Rubio et al. 2019a)
	The availability of FADD in the cytoplasm reduced in T-cell lymphoblastic lymphoma cells. Reduction of FADD phosphorylation that inversely correlates with the proliferation capacity and tumor aggressiveness. The reduction of FADD phosphorylation was negatively associated with the proliferation capacity and tumor aggressiveness.	Marin-Rubio et al. (Marin-Rubio et al. 2016)
	FADD was a direct target of miR-128a. FADD downregulation by miR-128a conferred Fas-resistance on Jurkat cells. FADD upregulation induced by miR-128a knockdown sensitized Jurkat/R cells to the Fas-mediated apoptosis.	Yamada et al. (Yamada et al. 2014)
Glioma	FADD was downregulated by ATRX via the H3K27me3 enrichment, resulting in the enhancement of PARP1 stabilization in TMZ resistant glioma cells.	Han et al. (Han et al. 2020)
	FADD overexpression induced apoptosis in about 85% of malignant glioma cells. The retroviral transfer of FADD gene significantly suppressed survival in malignant glioma cells by induction of apoptosis.	Kondo et al. (Kondo et al. 1998)
GBM	FADD overexpression inhibited proliferation and induced apoptosis in human GBM cells.	Wang et al. (Wang et al. 2017)
HCC	FADD mediated the role of OTULIN in preventing the development of chronic liver inflammation and HCC.	Verboom et al. (Verboom et al. 2020)
	FADD mediated the apoptosis of hepatocytes, thereby promoting the development of hepatitis and HCC in NEMO(LPC-KO) mice	Ehlken et al. (Ehlken et al. 2014)
	FADD mediated the role of truncated RIP3 (aa 224–518) in inducing apoptosis in human hepatocellular carcinoma cells QGY-7703.	Feng et al. (Feng et al. 2006)
	FADD expression was significantly correlated with cell apoptosis in HCC (p < 0.05). The positive rate of FADD expression in HCC is lower than that in adjacent normal tissues (p < 0.05).	Sun et al. (Sun et al. 2000)
Melanoma	The degradation of FADD was inhibited by ADT-OH via downregulating MKRN1. The tumor-specific delivery of FADD combined with low-dose ADT-OH administration significantly suppressed tumor growth and induced cell apoptosis in melanoma.	Cai et al. (Cai et al. 2020)
	The overexpression of FADD or truncated FADD (1-181 aa) induced apoptosis in B16F10 cells, The truncated FADD exhibited a more potent apoptotic effect than FADD. The tumor-targeted delivery of FADD or truncated FADD inhibited tumor growth by inducing apoptosis of tumor cells via activating caspase-dependent apoptotic pathway.	Yang et al. (Yang et al. 2016)
	FADD decreased FAK expression by upregulating miR-7a, thereby promoting cell migration in melanoma.	Liu et al. (Liu et al. 2016)
OC	FADD mediated the induction of apoptosis by certain chemotherapeutic drugs in OC cells.	Milner et al. (Milner et al. 2002)
CRC	Phosphorylated FADD interacted with MT2A and co-localized with MT2A mostly to nuclei and slightly to cytoplasm. The co-expression of Phosphorylated FADD and MT2A inhibited the apoptosis and promote proliferation in CRC cells.	Marikar et al. (Marikar et al. 2016)
	The overexpression of FADD mediated by adenovirus inhibited cell growth and enhanced apoptosis of SW480 cells and suppress growth of xenografts in mice.	He et al. (He et al. 2018)
	FADD mediated apoptosis of CRC cells synergistically induced by Smac mimetic and DOX.	Yang et al. (Yang et al. 2020b)
	Apigenin upregulated FADD and induced its phosphorylation, which may resulted in cell apoptosis and inhibition of tumor growth in CRC.	Wang et al. (Wang et al. 2011b)
	FADD overexpression enhanced 5-FU sensitivity in CRC mice model. Stable overexpression of FADD significantly improved apoptosis-inducing effects of 5-FU on CRC cells.	Yin et al. (Yin et al. 2010)
HNSCC	The immunohistochemical overexpression of FADD was significantly associated with worse OS (p < 0.001), DSS (p < 0.001), DFS ((p < 0.001), higher clinical stage (p = 0.005), and a large magnitude of effect with N + status (p < 0.001).	Gonzalez-Moles et al. (Gonzalez-Moles et al. 2020)
	High FADD expression was significantly associated with an increased rate of lymph node metastasis (p = 0.001), a shorter distant metastasis-free interval (p = 0.046).	Pattje et al. (Pattje et al. 2013)
	HNSCC patients with tumors that were strongly positive for cyclin D1 and FADD had reduced OS (p = 0.003 and p < 0.001), DSS (p = 0.039 and p < 0.001), and DFS (p = 0.026 and p < 0.001), respectively. FADD was a significant independent predictor of DSS and DFS.	Rasamny et al. (Rasamny et al. 2012)
OSCC	FADD gene copy number and protein expression are potential prognostic biomarkers and are closely associated with lymph node metastasis in OSCC patients (p < 0.001).	Chien et al. ((Chien et al. 2016)
Clear cell renal cell carcinoma	FADD was downregulated in clear cell renal cell carcinoma compared with that in normal tissues. FADD-mediated apoptosis may suppress carcinogenesis in clear cell renal cell carcinoma.	Xu et al. (Xu et al. 2009)
PC	FADD is required for PC cell proliferation and that it is overexpressed to varying degrees in a variety of PC cell types. FADD protected PC cells from drug-induced apoptosis, whereas FADD knockdown enhanced the sensitivity of drug-resistant cells to ADR-mediated apoptosis.	Zhang et al. (Zhang et al. 2017)

### FADD and cancer-related signaling pathways

The crosstalk of FADD with cancer-related signaling pathways, such as mitogen-activated protein kinase (MAPK) and nuclear factor-κB (NF-κB) signaling pathways, is considered a main mechanism through which FADD participates in the modulation of cancer progression. The MAPK signaling pathway is a conserved oncogenic pathway, and its aberrant activation has been shown to facilitate cancer progression by regulating multiple cellular processes, such as proliferation, apoptosis, and drug resistance (Liu et al. 2022 h). MAPKs mainly consist of three members of the c-Jun NH2 terminal kinase (JNK), extracellular stress regulated kinase (ERK), and p38 (Kciuk et al. 2022). Shimada et al. explored the effect of FADD phosphorylation mediated by JNK1 on drug sensitivity in prostate cancer (PCa) using cell models. Western blotting (WB) and flow cytometry (FCM) assays were performed. The results showed that FADD was phosphorylated by JNK1 in LNCaP and DU145 cells treated with paclitaxel (PTX), resulting in the enhancement of PTX sensitivity in PCa. Mechanistically, PTX administration increased MEK kinase 1 (MEKK1) levels in PCa cells, leading to the activation of JNK. Next, FADD was phosphorylated by JNK1 at Ser194, which further upregulated MEKK1 and enhanced downstream JNK1 activation, finally resulting in G2/M cell cycle arrest and apoptosis of PCa cells (Shimada et al. 2004). Tewari et al. examined the role of FADD in mediating miltefosine-induced apoptosis of glioma cells through WB and 3-(4,5-dimethylthiazol-2-yl)-5-(3-carboxymethoxyphenyl)-2-(4-sulfophenyl)-2 H-tetrazolium, inner salt assays. They discovered that the levels of phosphorylated ERK was significantly upregulated in U87MG and T98G cells treated with miltefosine, and activated ERK increased FADD expression, resulting in enhancement of glioma cell apoptosis induced by miltefosine. Consistent with this, the increase in FADD expression and cell apoptosis observed in U87MG and T98G cells treated with miltefosine was blocked in the presence of U0126 (an ERK inhibitor) (Tewari et al. 2008).

The NF-κB signaling pathway plays a vital role in regulating various biological processes, including cell differentiation, proliferation, and survival, and, most importantly, immune responses and inflammation (Chaithongyot et al. 2021). The dysregulation of NF-κB is closely associated with a variety of human diseases, particularly cancer (Liu et al. 2022a). Chaudhary et al. examined the effect of FADD on the NF-κB signaling pathway using a BC cell model. Luciferase reporter assay showed that low FADD concentration induced the activation of the NF-κB signaling pathway in MCF-7cells in a time- and dose-dependent manner, with caspase 8 or its homologs mediating this process (Chaudhary et al. 2000). FADD also exhibits the effect to inhibit NF-κB activation. Ranjan et al. performed luciferase reporter and p65 translocation assays and discovered that FADD overexpression blocked NF-κB activation induced by TNFα and translocation of p65 from the cytoplasm to the nucleus in MCF-7 cells and colorectal cancer (CRC) cell line HCT 116 (Ranjan and Pathak 2016b).The same team chemically conjugated purified FADD protein with cell permeable TAT (transactivator of transcription) peptide in another study, to deliver in cancer cells. Luciferase reporter and quantitative real-time polymerase chain reaction (qRT-PCR) assays showed that TAT-FADD significantly reduced the activation of NF-κB in HCT-116 cells, leading to the inhibition of anti-apoptotic genes, including *Bcl2*, *cIAPs*, *RIP1*, and *cFLIPL* (Ranjan et al. 2020). These studies suggest that FADD possesses double-edged role in regulating the NF-κB signaling pathway. The sufficient availability and concentration of FADD may be the cause of its differential roles in the activation of the NF-κB signaling pathway.

Taken together, these findings demonstrate that JNK1 induces G2/M cell cycle arrest and apoptosis in PCa cells by activating FADD, whereas ERK facilitates the apoptosis of glioma cells by upregulating FADD levels. FADD also exhibits double-edged effect in targeting the NF-κB signaling pathway in different cancer types. However, these studies have limitations. For instance, only cell models are used in these studies. Animal models and clinical samples should be utilized to validate these findings. In addition, there are various other cancer-related signaling pathways, such as the PI3K/AKT, Wnt/β-catenin, and STAT3 signaling pathways. Whether FADD can participate in cancer progression through synergistic action with these signaling pathways has not been reported. This issue should be included in future studies. Nevertheless, fully understanding the underlying mechanisms of FADD in the modulation of cancer-related signaling pathways may provide new insights into the development of effective therapeutic strategies for cancer patients.

### FADD and proliferation

One of the most remarkable hallmarks of cancer is the uncontrolled proliferation of tumor cells. The mechanisms involved in proliferation are extremely complicated and are yet to be fully understood (Liu et al. 2022e). Accumulating evidence suggests that FADD is a key regulator of cellular proliferation during cancer progression (Marin-Rubio et al. 2019a; Wang et al. 2017; Zhang et al. 2017). For example, Wang et al. performed qRT-PCR and WB assays and found that FADD was downregulated at both mRNA and protein levels in GBM tissues compared with that in normal brain tissues. Moreover, statistical analysis (SPSS 21.0 software) showed that FADD expression in stages III and IV patients was lower than those in stages I and II. Cell count kit-8 (CCK-8) analysis further revealed that FADD overexpression significantly inhibited the proliferation of the GBM cell line SHG44 (Wang et al. 2017). Zhang et al. conducted reverse transcription-quantitative polymerase chain reaction (RT-qPCR) and WB assays to examine the expression of FADD. FADD was significantly upregulated in the PC cell line Colo357 at both mRNA and protein levels. 3-(4,5-dimethylthiazol-2-yl)-2,5-diphenyl tetrazolium (MTT) assay showed that the knockdown of FADD significantly suppressed the proliferation of Colo357 cells (Zhang et al. 2017). Moreover, the data from fluorescence resonance energy transfer, MTT, Ki67 immunostaining, immunohistochemistry (IHC) assays demonstrated that Ser194-phosphorylated FADD was localized in the nucleus of tumor cells and induced proliferation in multiple types of cancer, including T-cell lymphoblastic lymphoma, CRC, and lung cancer (Bhojani et al. 2005; Marikar et al. 2016; Marin-Rubio et al. 2019a).

FADD could also mediate the effect of ncRNAs on proliferation in cancer. Yan et al. examined the effects H19 and miR-675 on GC cell proliferation using CCK-8 and colony formation assays and discovered that the overexpression of H19 and miR-675 facilitated the proliferation of SGC-7901 cells, whereas their knockdown had the opposite effects. The authors further explored the role of H19/miR-675 axis in GC using a nude mice model. A dramatic decrease was observed in tumor volume and weight in nude mice in which LV-shH19 infected SGC-7901 cells were injected. Co-infection with LV-miR675 partially reversed this tendency. Mechanistically, H19/miR-675 suppressed the caspase cleavage cascades (caspase 8 and caspase 3) by downregulating FADD, leading to the inhibition of the proliferation of SGC-7901 cells (Yan et al. 2017). In another study, Peng et al. explored the mechanism of circ 001418 involved in BCa cell proliferation using MTT and WB assays. Circ 001418 overexpression was found to facilitate proliferation of BCa cells by downregulating FADD and upregulating EphA2 and cytochrome c protein via sponging miR-1297 (Peng et al. 2021).

Collectively, these findings show that FADD can act as a tumor suppressor to inhibit cellular proliferation in several cancer types, such as GBM, CRC, and lung cancer. It can also facilitate proliferation in GC and BCa by serving as an oncoprotein. However, limitation still exists in these studies. For instance, almost all in vitro culture models are established using tumor cell lines. Primary cells originated from cancer patients should be used in future studies. In addition, the exact mechanisms of FADD involved in proliferation regulation are not fully elucidated. The upstream regulators and downstream effectors of FADD remain unclear. Therefore, uncovering regulatory network based on FADD involved in proliferation regulation should be an important direction in future studies.

### FADD and cell cycle

The cell cycle is a ubiquitous and complex process by which a cell duplicates its contents and divides into two genetically identical daughter cells. This process is tightly controlled by various cell cycle regulatory proteins, such as cyclin proteins, cyclin dependent kinases (CDKs), and mitotic checkpoint proteins (Roy et al. 2017). Dysregulation of the cell cycle has been shown to be a fundamental mechanism underlying carcinogenesis, making cell cycle regulators promising anti-tumoral therapeutic targets (Liu et al. 2022b). An increasing amount of evidence suggests that FADD is a key cell cycle regulator during cancer progression (Bowman et al. 2015; Chen et al. 2005; Matsuyoshi et al. 2006; Shimada et al. 2005). *Cyclin D1* and *Cyclin B1* are downstream target genes of NF-κB and are involved in regulating cell cycle process (Zhang et al. 2015). Chen et al. performed statistical analysis to evaluate the correlation between FADD and cyclin D1/B1 protein expression. They discovered that high FADD expression in lung cancer tissues was significantly associated with the overexpression of the cyclins D1 and B1. NF-κB TransAM kit and fluorescence-activated cell sorting assays were used to examine the role of FADD and p-FADD in cell-cycle progression. The results showed that phosphorylated FADD overexpression in Jurkat cells increased NF-κB activity and facilitated the accumulation of cells in the G2/M phase of the cell cycle (Chen et al. 2005). In another study, Bowman et al. analyzed the role of FADD phosphorylation in the regulation of cell cycle process in lung cancer. Co-immunoprecipitation (Co-IP) and mass spectrometry assays showed that CK1α phosphorylated FADD by directly interacting with it. Histopathology analysis of the H&E stain indicated that phosphorylated FADD mainly localized in the nucleus of lung cancer cells from a mice model. The data from WB and mass spectrometry assays further revealed that phosphorylated FADD was most abundant during the G2/M phase of the cell cycle, and it directly interacted with kinases (PLK1, AURA, and BUB1) that mediate the G2/M transition (Bowman et al. 2015). Moreover, Shimada et al. performed FCM analysis to detect the effect of phosphorylated FADD on cell cycle in primary cultures of normal epithelial cells and DU145 cells. They demonstrated that the induction of phosphorylated FADD in prostate epithelial cells resulted in cell cycle arrest at G2/M to protect against uncontrolled proliferation. FADD was more dephosphorylated in PCa cells than in normal epithelial cells, and dephosphorylated FADD promoted the cell cycle progression of PCa cells (Shimada et al. 2005). In addition, Matsuyoshi et al. conducted WB and FCM assays and discovered that FADD was phosphorylated by JNK in the ER-negative BC cell line MDA-MB-231 treated with PTX or tamoxifen. Phosphorylated FADD could induce cell cycle arrest at G2/M and inhibit cell proliferation (Matsuyoshi et al. 2006).

These findings demonstrate that FADD can be phosphorylated by a series of kinases (e.g., CKIα, PLK1, and BUB1). Phosphorylated FADD alters the expression of cell cycle regulatory proteins (e.g., Cyclin D1 and Cyclin B1) by activating NF-κB signaling pathway, thereby mediating the G2/M transition of cancer cells. Therefore, the phosphorylation status of FADD is crucial for the transition of cancer cells at the G2/M boundary. However, research on the role of FADD in regulating the cell cycle remains limited. For example, whether FADD can modulate cell cycle progression through other signaling pathways. In addition to phosphorylation, FADD can be modified by ubiquitination, SUMOylation, and GlcNAcylation. Whether these PTMs also play a role in cell cycle regulation during cancer progression. Future studies should focus on the clarification of these issues. Collectively, fully understanding the mechanisms of FADD in cell cycle regulation would help in the precise use of FADD-based therapeutics in particular types of cancer.

### FADD and apoptosis

Apoptosis induction is a major mechanism of many anti-cancer drugs in cancer treatment as apoptosis evasion results in therapy resistance for cancer patients (Singh and Lim 2022). Complex signaling pathways and a variety of regulators are involved in cell apoptosis during cancer progression. Thus, fully elucidating the underlying mechanisms involved in apoptosis may provide novel insights on the treatment of cancer. FADD is a well-known adaptor protein for DR-mediated extrinsic apoptosis (Zhou et al. 2022b). A large number of studies have shown that FADD is involved in cancer progression by regulating apoptosis (Hollomon et al. 2020; Mrkvova et al. 2021; Wang et al. 2017). For instance, Hollomorn et al. performed trypan blue exclusion and WB assays to examine the effect of FADD on TNFα-induced cell death. They discovered that FADD knockdown facilitated cell apoptosis in osteosarcoma LM7 or SaOS2 cells treated with TNFα. Further analysis revealed that the inhibition of NF-κB in wildtype LM7 cells significantly increased TNFα-induced apoptosis to similar levels observed in the FADD knockdown of LM7 cells, indicating that FADD plays a role in NF-κB pro-survival signaling (Hollomon et al. 2020). Wang et al. conducted qRT-PCR and WB to detect FADD expression and found that FADD was downregulated in GBM tissues and cell lines SC189, u251, and SHG44. The data from FCM analysis demonstrated that FADD overexpression significantly increased the apoptosis rate of SHG44 cells. Moreover, FADD-overexpressed GBM cells showed increased expressions of FADD, caspase-8, and Bax and decreased expressions of Bcl-2 (Wang et al. 2017). Mrkvová et al. identified molecules involved in cell death signaling pathways triggered by conventional anti-cancer drugs using Jurkat cell model with for necroptosis and/or apoptosis genes, created by the CRISPR-Cas9 editing system. The data from FCM analysis demonstrated that FADD was indispensable to the apoptosis induced by anti-cancer drugs (e.g., DOX and etoposide) in Jurkat cells. Consistent with this, Jurkat cells with knocked out FADD showed resistance to these drugs (Mrkvova et al. 2021). Furthermore, He et al. constructed a recombinant adenovirus containing FADD gene and explored the effect of FADD on cell growth and apoptosis using colon cancer cell and mice models. Results showed that FADD overexpression suppressed cell growth and enhance apoptosis in SW480 cells in vitro. Consistence with this, its overexpression also inhibited the growth of subcutaneous xenografts in the nude mice (He et al. 2018). .

Taken together, these studies demonstrate that FADD is a crucial regulator of apoptosis during cancer progression. Apoptosis can be classified into two categories: the extrinsic pathways mediated by DRs and intrinsic pathways mediated by mitochondria. FADD has been shown to be a key component for DR-mediated extrinsic apoptosis. In several studies, FADD was found to impair mitochondrial integrity and facilitate mitochondrial fragmentation (Choi et al. 2017; Ranjan and Pathak 2016a), indicating that FADD may participate in regulating mitochondria-mediated intrinsic apoptosis. Therefore, whether FADD is involved in the regulation of intrinsic apoptosis during cancer progression and the underlying mechanisms involved in this process should be further investigated in future studies. Although the detailed mechanisms of FADD involved in apoptosis are still not fully elucidated, it has exhibited great potential as a valuable target for cancer treatment.

### FADD and inflammation

Chronic inflammation is a well-known characteristic of cancer that substantially contributes to cancer initiation and progression (Murata 2018). Due to the high genetic stability of the cells responsible for cancer-associated inflammation, they are not subjected to the rapid emergence of drug resistance (Singh et al. 2019). Therefore, targeting inflammation is a promising strategy for cancer prevention and treatment. Accumulating evidence has pointed to the involvement of FADD in the regulation of inflammation during cancer progression. FADD participates and plays a vital role in most signalosome complexes, such as inflammasome and FADDosome (Henry and Martin 2017; Tummers et al. 2020). Ranjan et al. performed WB, RT-qPCR, and ELISA assays using colon cancer cell model to determine the role of FADD in inflammation regulation. They discovered that the delivery of FADD mediated by TAT in colon cancer HCT116 cells significantly suppressed the canonical NLRP3 inflammasome priming by downregulating NLRP3, thereby restricting the processing and secretion of proinflammatory IL-1β. Moreover, Luciferase reporter and qRT-PCR assays demonstrated that TAT-FADD inhibited the activation of NF-κB-induced proinflammatory priming in HCT116 cells (Ranjan et al. 2020). Henry et al. conducted co-IP, WB, ELISA assays using TRAIL-stimulated Hela cell model and showed that FADD formed the FADDosome complex by directly interacting with caspase-8 and RIPK1, thereby facilitating NF-κB activation and pro-inflammatory cytokine production (e.g., IL-6, IL-8, and MCP-1) (Henry and Martin 2017). In another study, Hartwig et al. validated these results in human lung adenocarcinoma (ADC) A549 cells. They examined secretion of proinflammatory cytokines by ELISA assay and found that that FADD mediated the promotion of TRAIL upon secretion of proinflammatory cytokines IL-8 and MCP-1 in human lung adenocarcinoma (ADC) A549 cells. Consistent with this, FADD knockdown inhibited NF-κB activation and inflammatory cytokine production in A549 cells in response to TRAIL stimulation (Hartwig et al. 2017).

Collectively, these findings show that FADD exerts proinflammatory effects in multiple types of cancer, including CRC, CC, and ADC. However, FADD can also contribute to anti-inflammatory responses of some non-tumor cell lines (Hu et al. 2000; Schaub et al. 2000). For instance, FADD overexpression in MEFs was found to suppress LPS-induced NF-κB activation but promote LPS-induced IFNβ production (Brennan et al. 2019), which means that FADD may possess great potential to play an anti-inflammatory role in some other cancer types. This hypothesis should be further tested in future studies. In addition, there are limitations in these studies as only cell models were used in exploring the role of FADD in inflammation. Animal models and patient samples should be applied in future studies to validate these findings. Nevertheless, in-depth investigations are required to elucidate the detailed mechanisms of FADD involved in inflammation regulation during cancer progression, which may provide new insights into the development of FADD-based therapeutic strategies for cancer patients.

### FADD and drug resistance

The development of drug resistance to chemotherapy continues to be one of the important reasons for treatment failure and patient death in cancer (Liu et al. 2022d). The mechanisms underlying drug resistance mainly include tumor heterogeneity, some cellular-level changes, and genetic factors, though not fully understood (Nussinov et al. 2021). It has been reported that FADD is a crucial regulator of drug resistance during cancer progression (Han et al. 2020; Mrkvova et al. 2021; Pang et al. 2019). For instance, in our previous study, FADD was found to mediate the inhibitory effect of miR-633 on drug resistance in GC cell models. The data from WB, qRT-PCR and TUNEL assays demonstrated that FADD was downregulated by miR-633, which significantly enhanced the apoptosis of SGC-7901 cells induced by DOX/CDDP (Pang et al. 2019). Han et al. showed that FADD plays a vital role in temozolomide (TMZ)-mediated drug resistance in glioma. They performed immunofluorescence assay using glioma cell model and discovered that FADD overexpression increased temozolomide (TMZ)-induced DNA damage in glioma cells, whereas FADD knockdown decreased TMZ-induced DNA damage in ATRX knockout glioma cells. Furthermore, the data from WB, qRT-PCR, and immunofluorescence assays revealed that FADD expression was inhibited in TMZ-resistant glioma cells by ATRX by promoting the trimethylation of histone H3K27 in the *FADD* promoter region. The downregulation of FADD mediated by ATRX promoted PARP1 stabilization, resulting in the enhancement of DNA damage repair (Han et al. 2020). Moreover, Zhang et al. conducted RT-qPCR, WB, and MTT assays to analyze the effect of FADD on drug resistance in PCa cell models. They found that FADD was upregulated to varying degrees in various types of PCa cells. This results in differing levels of drug resistance in PCa cells. Furthermore, drug resistance is positively associated with FADD expression. The data from Annexin V immunostaining analysis revealed that FADD could protect PCa cells from Adriamycin-induced apoptosis (Zhang et al. 2017).

These studies strongly suggest that FADD plays a double-edged role in the development of cancer drug resistance. FADD can inhibit drug resistance in glioma cells through enhancing DNA damage induced by TMZ. It can also enhance drug resistance in GC and AML cells through suppressing apoptosis induced by chemotherapeutic agents (e.g., DOX and Adriamycin). However, the exact effects of FADD on drug resistance in cancer are still unclear. For instance, whether FADD possesses the same effect in mediating one anti-cancer drug in different cancer types. Whether FADD have the same role in mediating multiple anti-cancer drugs in one cancer. Moreover, multiple mechanisms, such as excessive drug efflux, alteration of CSC characteristics, and autophagy dysregulation, can result in the development of drug resistance in cancer. Whether FADD is involved in cancer drug resistance by regulating these processes. All these concerns should be addressed in future studies. Taken together, further studies on the mechanisms of FADD involved in drug resistance in distinct types of cancer will be of great benefit to the development of FADD-based therapeutic strategies for patients demonstrating a poor response to chemotherapy.

**Fig. 3:**
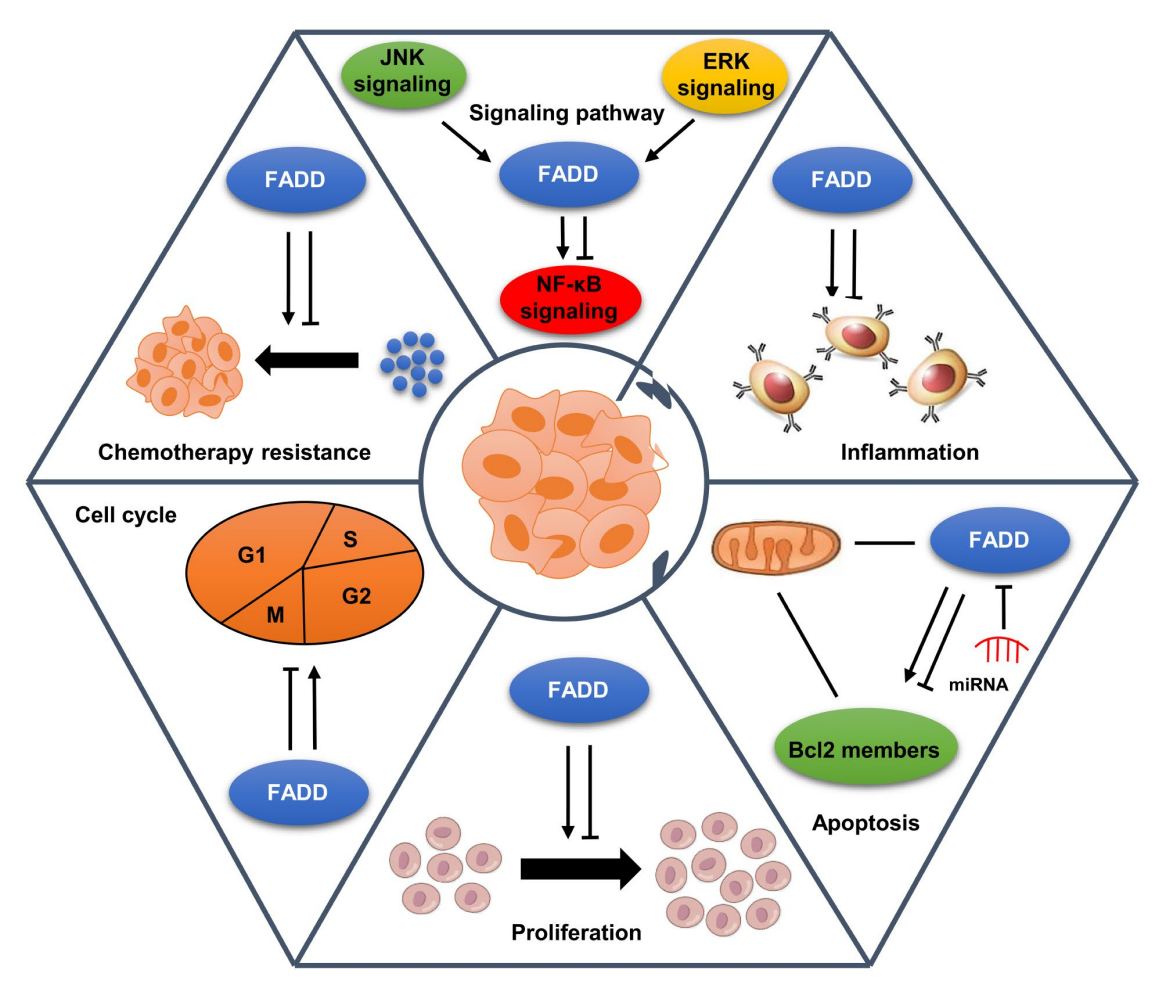
Role of FADD in cancer progression. FADD dysregulation contributes to cancer progression by various mechanisms, including crosstalk with oncogenic signaling pathway, induction of inflammation, inhibition of apoptosis, promotion of proliferation, modulation of cell cycle, and enhancement of drug resistance

In summary, FADD may play the dual role of tumor suppressor or oncoprotein in cancer progression. FADD dysregulation contributes to cancer progression by influencing many aspects of cancer cell behavior, including proliferation, apoptosis, cell cycle, inflammation, and drug resistance (Fig. 3). The double-edged effect of FADD on the development of cancer drug resistance may depend on its diverse functions.

## Clinical applications of FADD in cancer treatment

### FADD as a prognostic biomarker in cancer

Prognostic assessment is a core link in the management of cancer patients. Accurately assessing a prognosis can help judge the efficiency of a current therapeutic strategy and adjust a proper treatment plan for cancer patients in a timely manner (Simmons et al. 2017). However, patient outcomes continue to be poor due to the lack of effective evaluation methods. Biomarkers are considered a promising tool in the early diagnosis, prognostic assessment, and treatment of cancer patients (Ao et al. 2020; Lu et al. 2021). Some classical biomarkers (e.g., CEA, AFP, and CA72-4) have been clinically applied, but their unideal specificity and sensitivity restrict their further utilization (Liu et al. 2022c). Therefore, there is an urgent need in cancer treatment for the exploration of reliable biomarkers with high specificity and sensitivity. FADD has exhibited great potential as a prognostic biomarker for cancer patients due to its aberrant expression pattern in different types of cancer (Table 3).

**Table 3:** FADD as a prognostic biomarker in cancer

Cancer types		Clinical values	References
HNSCC	FADD overexpression was significantly associated with worse OS (p < 0.001), DSS (p < 0.001), and DFS (p < 0.001).		Gonzalez-Moles et al. (Gonzalez-Moles et al. 2020)
	A 13-gene signature consist of *FADD*, *GABARAPL1*, *ITGA3*, *USP10*, *ST13*, *MAPK9*, *PRKN*, *IKBKB*, *ITPR1*, *TP73*, *MAP2K7*, *CDKN2A*, and *EEF2K* was an independent prognostic factor (p < 0.001) for NSCLC patients. The 13-gene signature significantly stratified HNSCC patients into high- and low-risk groups in terms of OS (p < 0.0001). The AUC vale were significant for both the TCGA (0.685) and GEO (0.928).		Ren et al. (Ren et al. 2021)
	A 3-gene signature consist of *FADD*, *EGFR*, and *PARK2* was an independent prognostic biomarker. ROC analyses revealed high predictive accuracy and sensitivity of the 3-gene signature.		Jiang et al. (Jiang et al. 2021)
	A 6-gene signature consist of *FADD*, *EGFR*, *HSPB8*, *PRKN*, *CDKN2A*, and *ITGA3* was an independent prognostic biomarker (AUC = 0.709) for NSCLC patients.		Yang et al. (Yang et al. 2020a)
	Lower FADD expression was significantly correlated with better OS (p = 0.0001) and DFS (p = 0.0006) in HNSCC patients with lymph node metastasis.		Fan et al. (Fan et al. 2013)
	A 7-gene signature consist of *FADD*, *ITGA3*, *CDKN2A*, *NKX2-3*, *BAK1*, *CXCR4*, and *HSPB8* was proved to be effective in predicting the survival rate of HNSCC patients (p = 8.409 × 10^− 6^).		Jin et al. (Jin and Qin 2020)
NSCLC	Lung ADC patients with overexpression of FADD exhibited significantly lower OS rates (p = 0.033). FADD overexpression was an independent poor prognostic biomarker for patients with surgically resected lung ADC (p = 0.027).		Chen et al. (Chen et al. 2021)
ESCC	A 13-gene signature consist of *FADD*, *PARP1*, *ITGA6* was an independent prognostic factor for ESCC patients. This signature effectively stratified patients in both discovery and validation cohorts by OS (p = 5.162E-8 and p = 0.052, respectively).		Cui et al. (Cui et al. 2021)
BC	FADD expression was significantly associated with T stage (P = 0.046) in BC patients. The combination of *FADD*, *PPFIA1*, and *TMEM16A* gene expressions was significantly correlated with perineural invasion (p = 0.022). Moreover, the combination of the three gene expressions was closely associated with DFS (p = 0.034).		Choi et al. (Choi et al. 2014)
Lymphoma	The phosphorylation of FADD (Ser194) was identified as a prognostic biomarker in T-cell lymphoblastic lymphoma.		Marin-Rubio et al. (Marin-Rubio et al. 2019a)
	Low or absent expression of the FADD in leukemic cells was identified as a poor independent prognostic biomarker.		(Tourneur et al. 2004)
Lung cancer	A 6-gene signature consist of *FADD*, *EIF4EBP1*, *TP63*, *BNIP3*, *ATIC*, and *ERO1A* was identified as an independent prognostic biomarker for lung adenocarcinoma and lung squamous cell carcinoma.		Zhu et al. (Zhu et al. 2020)
OSCC	FADD gene copy number and protein expression were identified as potential prognostic biomarkers in patients with OSCC. Patients with both FADD copy number amplification and high protein expression exhibited the shortest DFS (p = 0.074 and p = 0.002) and OS (p = 0.011 and p = 0.027).		Chien et al. (Chien et al. 2016)

Chen et al. showed that lung ADC patients with FADD overexpression had a significantly lower overall survival (OS) rate (p = 0.033). Multivariate analysis further confirmed that FADD upregulation was an independent poor prognostic biomarker for patients with surgically resected lung ADC (p = 0.027) (Chen et al. 2021). González-Moles et al. performed a meta-analysis to evaluate the prognostic significance of FADD upregulation in HNSCC. They found that elevated FADD expression was significantly associated with poor OS (p < 0.001), disease-specific survival (DSS) (p < 0.001), and disease-free survival (DFS) (p < 0.001) (Gonzalez-Moles et al. 2020). *FADD* gene copy number amplification can also be used as a potential prognostic biomarker. For example, Eytan et al. discovered that HNSCC harbored the most frequent genomic amplification of FADD, affecting approximately 30% of patients with poor prognosis (Eytan et al. 2016). Chien et al. showed that both FADD gene copy number amplification and elevated protein expression were significantly correlated with lymph node metastasis in OSCC patients (p < 0.001). OSCC patients with both *FADD* gene copy number amplification and elevated protein expression showed the shortest DFS (p = 0.074 and p = 0.002) and OS (p = 0.011 and p = 0.027) (Chien et al. 2016). In multiple studies, phosphorylated FADD was identified as a promising prognostic biomarker in distinct types of cancer, including T-cell lymphoblastic lymphoma, PCa, and glottic squamous cell carcinoma (Marin-Rubio et al. 2019a; Matsumura et al. 2009; Schrijvers et al. 2012). In addition, the combination of *FADD* with other genes can be used to predict cancer treatment prognosis (Cui et al. 2021; Jiang et al. 2021; Yang et al. 2020a). For example, Choi et al. demonstrated that FADD expression was significantly associated with the T stage (p = 0.046) in patients with invasive ductal carcinoma of the breast. Moreover, the combination of *FADD*, *TMEM16A*, and *PPFIA1* expressions showed significant associations with DFS (P = 0.034) (Choi et al. 2014). Collectively, these studies strongly suggest that FADD possesses great potential as a valuable prognostic biomarker for cancer patients. However, large patient cohorts should be included in future studies to further confirm the application of FADD as a prognostic biomarker in clinics.

### FADD as therapeutic target in cancer

FADD has been shown to participate in cancer progression by altering various cellular processes, including proliferation, apoptosis, cell cycle, inflammation, and drug resistance. High FADD expression contributes to the progression of multiple types of cancer, such as PC, OSCC, HNSCC, and BC, indicating that FADD acts as an oncoprotein in these cancers (Chien et al. 2016; Gonzalez-Moles et al. 2020; Zhang et al. 2017; Zhou et al. 2022a). Thus, a screening or synthesis of novel agents targeting FADD may be an effective therapeutic strategy in these cancers. By contrast, low FADD expression has been observed in several certain types of cancer, such as thymic lymphoma, AML, and GBM, suggesting that FADD serves as a tumor suppressor in these cancers (Newton et al. 2000; Tourneur et al. 2004; Wang et al. 2017). Therefore, the upregulation of FADD in these cancers represents another promising strategy in cancer treatment. In addition, a series of ncRNAs, such as miR-633, H19, and circ 001418, have been identified as upstream regulators of FADD in cancer progression (Pang et al. 2019; Peng et al. 2021; Yan et al. 2017). Targeting these ncRNAs is also an attractive strategy in overcoming cancer. Research on novel agents targeting FADD in cancer treatment remains in the pre-clinical stage. Further studies are required to translate FADD-based therapeutic strategies into clinical utilization in treatment.

## Conclusion and perspective

FADD plays a vital role in normal physiological processes by regulating many aspects of cell behavior, such as apoptosis, proliferation, autophagy, and necroptosis. Its expression and activity are tightly controlled through a synergistic regulatory network involving DNA amplification, promoter methylation, gene mutation, TFs, ncRNAs, and PTMs. Therefore, the dysregulation of FADD is closely associated with the initiation and development of many diseases, particularly cancer. FADD dysregulation contributes to cancer progression through various mechanisms, including cross-talking with cancer-related signaling pathways, inhibiting cell apoptosis, facilitating proliferation, disordering cell cycle, inducing inflammation, and enhancing drug resistance. These characteristics endow FADD with great potential as a valuable therapeutic target for a wide range of cancers. Thus, the screening or synthesis of novel agents targeting FADD may be an effective therapeutic strategy for cancer patients. However, current studies are still in the stage of basic and preclinical research. Clinical trials with large patient cohorts should be included as an important direction in future studies. The differential expression of FADD in distinct cancer types also offers an exciting possibility for the development of cancer-tissue-specific treatment plans. For example, FADD is highly expressed in PC, OSCC, BC, and HNSCC, and it promotes the progression of these cancers by serving as an oncoprotein. Targeting oncogenic FADD in these cancer types could be an effective therapeutic strategy for patients. Conversely, FADD is lowly expressed in thymic lymphoma, AML, and GBM, and its overexpression inhibits the progression of these cancers. The upregulation of tumor-suppressive FADD may represent an effective way to overcome these cancer types. In addition, FADD overexpression has been found to enhance drug resistance in a variety of cancers (e.g., GC and PCa), indicating the feasibility of targeting FADD to enhance efficiency in chemotherapy (Pang et al. 2019; Zhang et al. 2017). Accurately evaluating prognoses is a core link in cancer treatment for patients to adjust their therapeutic strategies and prolong their lifespan. The aberrant expression of FADD has been shown to be significantly associated with some pathological features of cancer patients (e.g., OS, DSS and DFS), strongly indicating its potential value as a prognostic biomarker in cancer treatment (Chen et al. 2021; Chien et al. 2016; Gonzalez-Moles et al. 2020). However, there are still some unsolved challenges regarding the strategy of targeting FADD in cancer patients due to the essential role that FADD plays in normal physiological processes. For instance, targeting FADD may cause unexpected abnormal pathological reactions in cancer patients. Nevertheless, FADD has exhibited great potential as a therapeutic target and/or prognostic biomarker in cancer treatment. Continued in-depth research into its various roles in cancer progression may be of great benefit to the development of FADD-based therapeutic strategies for cancer patients.

## Data Availability

Not applicable.

## Abbreviations

FADD: Fas-associated protein with death domain
DD: death domain
DR: death receptor
NSCLC: non-small cell lung cancer
HCC: hepatocellular carcinoma
DED: death effector domain
TNF: tumor necrosis factor
RIPK: receptor-interacting protein kinase
ATG5: autophagy related-protein 5
NLS: nuclear localization signal
NES: nuclear export signal
DISC: death-inducing signal complex
cFLIP: cellular FLICE (caspase 8)-inhibitory protein
MLKL: mixed lineage kinase domain-like protein
IFN: interferon
Cryo-EM: cryogenic electron microscopy
tDED: tandem DED
TF: transcription factor
ncRNA: non-coding RNAs
PTM: post-translational modifications
MEFs: murine embryonic fibroblasts
BC: breast cancer
BCa: bladder cancer
OSCC: oral squamous cell carcinoma
LUSC: lung squamous cell carcinoma
OC: ovarian cancer
ESCC: esophageal squamous cell carcinoma
HNSCC: head and neck squamous cell carcinoma
CC: cervical cancer
WB: western blotting
FCM: flow cytometry
qRT-PCR: quantitative real-time polymerase chain reaction
CCK-8: cell count kit-8
RT-qPCR: reverse transcription-quantitative polymerase chain reaction
MTT: 3-(4,5-dimethylthiazol-2-yl)-2,5-diphenyl tetrazolium
Co-IP: co-immunoprecipitation
HIF-1α: hypoxia-inducible factor-1α
BRCA1: BC susceptibility gene 1
HRE: hypoxia-responsive element
mRNA: messenger RNA
miRNA: microRNA
lncRNA: long non-coding RNA
circRNA: circular RNA
piRNA: piwi-interacting RNA
3′UTR: 3′-untranslated region
GC: gastric cancer
DOX: doxorubicin
CDDP: cisplatin
NP: nucleus pulposus
CK: casein kinase
PLK: polo kinase
AURA: Aurora-A kinase
MKRN1: makorin ring finger protein 1
CHIP: C terminus HSC70-interacting protein
LUBAC: linear ubiquitin chain assembly complex
Drp1: dynamin-related protein 1
GlcNAc: N-acetylglucosamine
AML: acute myeloid leukemia
GBM: glioblastoma
PC: pancreatic cancer
MAPK: mitogen-activated protein kinase
NF-κB: nuclear factor-κB
JNK: c-Jun NH2 terminal kinase
ERK: extracellular stress regulated kinase
PTX: paclitaxel
PCa: prostate cancer
MEKK1: MEK kinase 1
TAT: trans-acting activator of transcription
CRC: colorectal cancer
CDK: cyclin dependent kinases
ADC: adenocarcinoma
TMZ: temozolomide
OS: overall survival
DSS: disease-specific survival
DFS: disease-free survival
